# Predictive markers of clinical outcome in the GRMD dog model of Duchenne muscular dystrophy

**DOI:** 10.1242/dmm.016014

**Published:** 2014-09-26

**Authors:** Inès Barthélémy, Fernanda Pinto-Mariz, Erica Yada, Loïc Desquilbet, Wilson Savino, Suse Dayse Silva-Barbosa, Anne-Marie Faussat, Vincent Mouly, Thomas Voit, Stéphane Blot, Gillian Butler-Browne

**Affiliations:** 1 Université Paris-Est, Ecole Nationale Vétérinaire d’Alfort, UPR de Neurobiologie, 94704 Maisons-Alfort, France.; 2 Université Pierre et Marie Curie-Paris 06 UM76, INSERM U974, CNRS UMR 7215, Institut de Myologie, Paris, France.; 3 Institute of Pediatrics/Federal University of Rio de Janeiro (IPPMG/UFRJ), Rio de Janeiro 21941-912, Brazil.; 4 Laboratory of Thymus Research, Oswaldo Cruz Foundation, Rio de Janeiro 21040-360, Brazil.; 5 Université Paris-Est, Ecole Nationale Vétérinaire d’Alfort, Unité D’Épidémiologie clinique et de Biostatistique, 94704 Maisons-Alfort, France.; 6 CNRS UMR 7179, MNHN, Brunoy 91800, France.; 7 Department of Clinical Research, National Cancer Institute (INCA), Rio de Janeiro 20230-130, Brazil.; 8 Université Pierre et Marie Curie-Paris 06 IFR 65 Saint-Antoine, Paris 75005, France.

**Keywords:** GRMD, DMD, Dystrophin, Dog, Predictive biomarker, Lymphocyte, CD49d, Gait analysis, Accelerometry

## Abstract

In the translational process of developing innovative therapies for DMD (Duchenne muscular dystrophy), the last preclinical validation step is often carried out in the most relevant animal model of this human disease, namely the GRMD (Golden Retriever muscular dystrophy) dog. The disease in GRMD dogs mimics human DMD in many aspects, including the inter-individual heterogeneity. This last point can be seen as a drawback for an animal model but is inherently related to the disease in GRMD dogs closely resembling that of individuals with DMD. In order to improve the management of this inter-individual heterogeneity, we have screened a combination of biomarkers in sixty-one 2-month-old GRMD dogs at the onset of the disease and *a posteriori* we addressed their predictive value on the severity of the disease. Three non-invasive biomarkers obtained at early stages of the disease were found to be highly predictive for the loss of ambulation before 6 months of age. An elevation in the number of circulating CD4^+^CD49d^hi^ T cells and a decreased stride frequency resulting in a reduced spontaneous speed were found to be strongly associated with the severe clinical form of the disease. These factors can be used as predictive tests to screen dogs to separate them into groups with slow or fast disease progression before their inclusion into a therapeutic preclinical trial, and therefore improve the reliability and translational value of the trials carried out on this invaluable large animal model. These same biomarkers have also been described to be predictive for the time to loss of ambulation in boys with DMD, strengthening the relevance of GRMD dogs as preclinical models of this devastating muscle disease.

## INTRODUCTION

Duchenne muscular dystrophy (DMD) is the most common genetic muscular dystrophy, affecting 1 in 3500–5000 male births. It is caused by mutations in the dystrophin gene, leading to functional loss or absence of the protein at the sarcolemma of muscle fibers (Deconinck and Dan, 2007). Individuals with DMD exhibit progressive muscle weakness leading to the permanent use of a wheelchair in young adolescents, and to respiratory and heart failure in young adults. The disease course, although following these constant patterns, is highly variable between affected individuals, a striking example being the age for the loss of ambulation, which can range from 6 to 15 years (Flanigan et al., 2013; Ricotti et al., 2013). Several recent studies have focused on the phenotypic variability, and have tried to define stratification criteria (Desguerre et al., 2009a; Humbertclaude et al., 2012) and identify modulators of disease severity (Flanigan et al., 2013; Pegoraro et al., 2011) in order to ultimately increase the success of clinical trials aiming to provide a therapeutic solution to treat or alleviate DMD.

In order to develop new strategies of treatment, different animal models have been used. In this context, the dystrophin-deficient dog, notably the Golden Retriever muscular dystrophy (GRMD) dog, represents a “translational bridge between mice and humans” (Duan, 2011), because it mimics more closely the human disease than other existing mammalian models of dystrophin deficiency (Valentine et al., 1988). GRMD dogs harbor a mutation in the dystrophin gene, and display dystrophic muscle lesions, inflammatory foci, progressive fibrosis and fatty infiltration, early locomotor impairment and premature death due to respiratory or cardiac failure. A wide inter-individual variability also figures among the numerous similarities shared by canine and human diseases. At first glance, this inter-individual variability can be seen as a major obstacle in the evaluation of therapies at the preclinical stage (Banks and Chamberlain, 2008; Willmann et al., 2009), but this model could also be relevant in discovering modulatory factors and modifier genes for this disease. Taking advantage of this inter-individual heterogeneity to identify predictive markers of the clinical evolution could help to increase the robustness of preclinical trials on the one hand, and unveil mechanisms underlying variations in disease expression on the other hand.

Using this approach, in a cohort of 74 individuals with DMD, we have shown that the relative number of circulating T cells with a higher membrane expression level of CD49d (T^+^/CD49d^hi^) – the α4 chain of the integrin VLA-4 – correlate with the progression and prognosis of the disease (F.P.-M., W.S., S.D.S.-B., V.M., T.V., G.B.-B. et al., unpublished data). This subset of T cells has been shown to be more prominent in individuals with DMD and, more importantly, these T-cell subpopulations have an enhanced migration potential (Pinto-Mariz et al., 2010) and participate to the damaging inflammatory infiltrate in DMD muscles (F.P.-M., W.S., S.D.S.-B., V.M., T.V., G.B.-B. et al., unpublished data). Consequently, CD49d can be used in individuals with DMD not only as a muscle inflammation biomarker, reflecting the progression of the disease, but can also serve as a predictive biomarker (F.P.-M., W.S., S.D.S.-B., V.M., T.V., G.B.-B. et al., unpublished data). Another study has shown that timed motor performances (time to walk 30 feet) in individuals with DMD were also accurate to predict the time before loss of ambulation (McDonald et al., 1995).

In GRMD dogs, no such predictive marker has been identified. In this study, we have assessed the predictive value of the blood lymphocyte subpopulations expressing high levels of CD49d, and of gait abnormalities at the onset of the disease. We were successful in identifying the first predictive biomarkers in GRMD and we provide a set of three ready-to-use tests that should significantly improve the quality of the therapeutic trials carried out in GRMD dogs.

**TRANSLATIONAL IMPACT**

**Clinical issue**

Duchenne muscular dystrophy (DMD) is a recessive X-linked genetic disease that involves the whole striated and cardiac musculature of the body and that leads to loss of ambulation in young teenagers and death by the third decade of life. No curative solution is currently available for this disease. Canine models of DMD, including GRMD (Golden Retriever muscular dystrophy), closely mimic the human condition and are particularly relevant to address preclinical questions. Among the similarities between canine and human diseases, the inter-individual clinical heterogeneity is a prominent feature; however, this makes results from preclinical and clinical studies difficult to decipher. In this study, motor and lymphocytic biomarkers have been evaluated at an early stage of the canine disease. In addition, their ability to correlate with the severity and progression of the disease has been assessed to develop a predictive test that could improve the translational value of preclinical studies.

**Results**

A population of 61 GRMD dogs was evaluated at 2 months of age (clinical onset) and was subsequently divided into two subgroups according to the occurrence of ambulation loss before 6 months of age (severe form, which affected one fourth of the dogs in the study). An elevated proportion of circulating lymphocytes expressing high levels of the integrin CD49d was found predictive of the severe form. In the same way, low spontaneous gait speed and stride frequency were strongly associated with the loss of ambulation. Interestingly, these markers have also been shown to be predictive of the time to loss of ambulation in individuals with DMD. The reliability of a predictive test based on these non-invasive and simply obtained markers was assessed, demonstrating that they can be used to classify dogs based on their future clinical evolution, with good specificity and sensitivity values.

**Implications and future directions**

This study provides ready-to-use tests that can significantly advance the predictive value of preclinical screenings for DMD candidate therapies. Indeed, the use of the investigated biomarkers can be realistically envisioned, either as inclusion criteria, or as covariates in the analysis of preclinical results. Beyond the possibility to significantly improve the preclinical phase of clinical studies, the fact that these predictive biomarkers are shared between dogs and humans reinforces the translational relevance of this canine model of DMD. Indeed, clinical heterogeneity could be managed in the same way during preclinical and clinical steps, optimizing the translation of results. Future experiments should focus on the mechanisms underlying the clinically relevant variation of these biomarkers, and could lead to the identification of therapeutic targets for DMD.

## RESULTS

### Loss of ambulation affects one-third of GRMD dogs, and occurs before 6 months of age

**Fig. 1.**
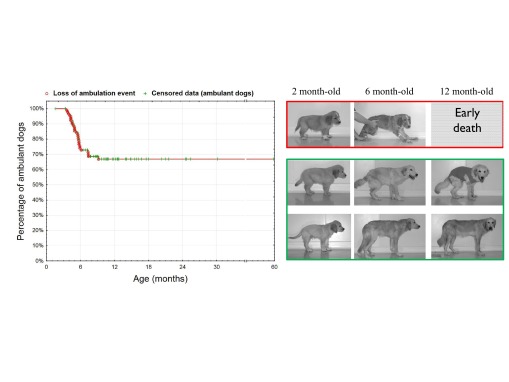
**Loss of ambulation in GRMD dogs and classification in two clinical forms as a function of the occurrence of this clinical event.** In the left panel, the curve represents a Kaplan-Meier curve that shows the percentage of ambulant dogs according to age (*n*=94 GRMD dogs; S.B. and I.B., unpublished data). Loss-of-ambulation events are represented in red, and censored data (either age of still alive and ambulant dogs at the end of the analysis period, or age at death of ambulant dogs) in green. The loss of ambulation occurs in about one third of the dogs, mainly before 6 months of age (85% of the dogs with a loss of ambulation). A classification into two sub-phenotypes is proposed and illustrated on the right panel: severe form (red frame) are opposed to moderate form (green frame) and are characterized by a loss of ambulation before 6 months of age and an early death (euthanasia owing to the permanent recumbence).

Inter-individual heterogeneity has been widely described in GRMD dogs but few clinical stratifications have been proposed (Ambrósio et al., 2009). In our hands the loss or maintenance of ambulation is the only clear dichotomic parameter that can define subgroups. This loss of ambulation occurs in one out of three GRMD dogs, in 85% of cases before the age of 6 months (Fig. 1). These animals develop severe contractures and an accelerated progression of their gait impairment (Barthélémy et al., 2011), leading to the loss of ambulation and permanent recumbence by 6 months of age. We defined these particularly devastating clinical presentations (i.e. loss of ambulation before the age of 6 months) as the ‘severe form’. In contrast, the remaining GRMD dogs were usually able to maintain ambulation until their death and were classified as having a ‘moderate form’ of the disease (i.e. dogs with either a loss of ambulation after the age of 6 months or no loss of ambulation).

The aim of the present study is to assess whether blood and gait parameters could predict the evolution towards one of these two clinical forms, at an early stage of the disease. The age of 2 months was targeted because this is the age of weaning for the puppies and is also considered as the clinical onset of the disease. From a practical point of view, it corresponds to the earliest time point for the longitudinal studies performed in GRMD dogs (Barthélémy et al., 2011; Thibaud et al., 2012) and a time after which most of the preclinical trials are designed to begin.

A total of sixty-one 2-month-old GRMD dogs were enrolled in the study, 15 of which evolved towards the severe form. Thirty-five dogs were enrolled in the blood study (severe forms *n*=9/35) and 57 dogs in the motor study (severe forms *n*=15/57). The proportion of the severe form in each group was consistent with the usual observations that have been made in this colony. Two dogs lost ambulation after 6 months of age (at, respectively, 7.33 and 7.37 months of age), and were thus categorized as moderate forms. Their late loss of ambulation was taken into account in the survival analysis.

### The proportion of CD4^+^ T cells with a CD49d^hi^ surface antigen profile is increased in 2-month-old GRMD dogs with a severe clinical form

In a first set of experiments, we evaluated the membrane expression of CD49d (the α4 chain of the VLA-4), comparing the two different groups of GRMD dogs with healthy controls at 2 months of age. We found a significantly higher relative number of CD4 T cells expressing high levels of CD49d (CD4^+^CD49d^hi^ T cells) in the blood of dogs with the severe form (SF) of the disease.

**Fig. 2.**
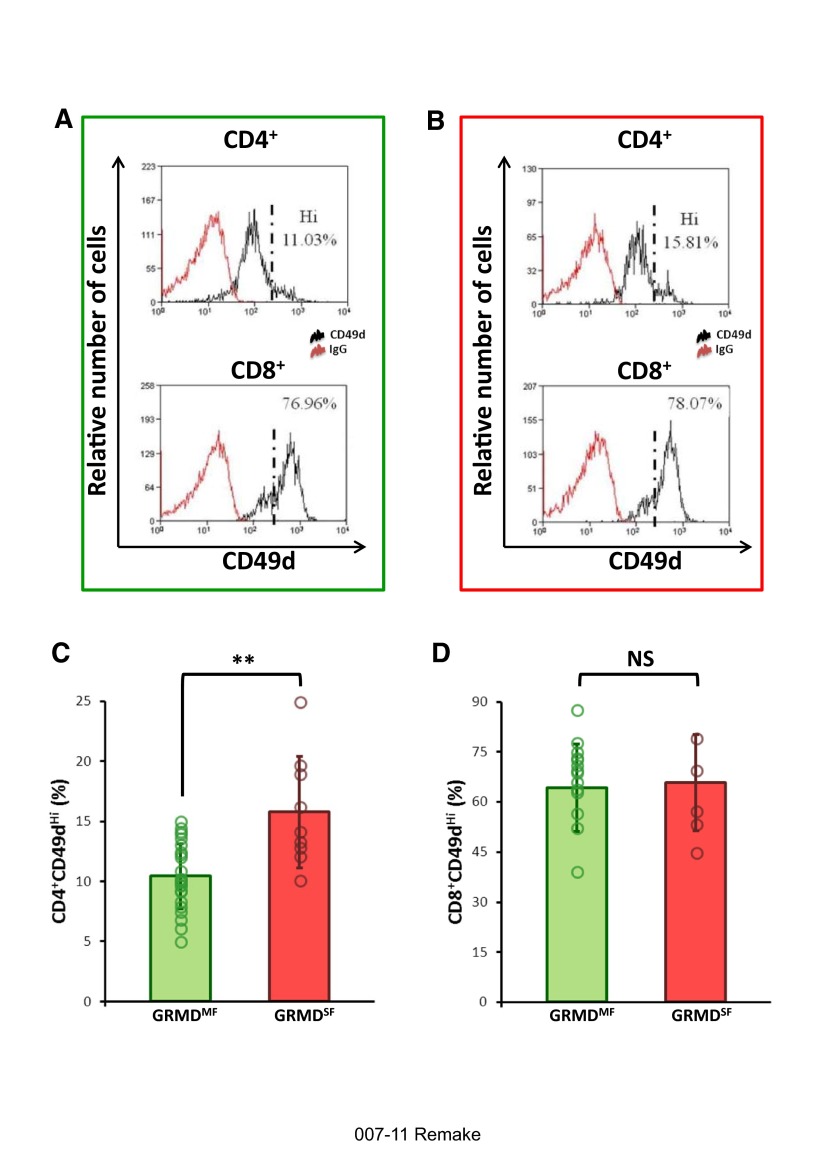
**Analysis of the expression of CD49d on circulating T cells, and results in 2-month-old dogs as a function of their clinical form.** (A,B) Typical CD49d cytometric profiles from a moderate form (A, green frame) and a severe form (B, red frame). Upper profiles show the profile of CD49d expression in CD4^+^ cells, and lower profiles in CD8^+^ cells. The population expressing high levels of CD49d is located to the right of the black dotted line drawn on the graphs. (C,D) Graphs represent the comparison of the percentages of CD4^+^CD49d^hi^ and CD8^+^CD49d^hi^ cells obtained in 2-month-old GRMD dogs with would-be moderate forms (GRMD^MF^; in green) versus severe forms (GRMD^SF^; in red). The height of the histograms provides the mean, and the error bars indicate ± 1 s.d. Individual values have been superimposed on the histograms and are represented by empty circles. **Significant difference (*P*<0.01); NS, not significant.

Otherwise, no significant differences were observed in the relative numbers of CD4^+^CD49d^hi^ T cells between the dogs with the moderate form (MF) and healthy controls, nor between the relative numbers of CD8^+^CD49d^hi^ T cells in the blood of GRMD and control dogs. In conclusion, a high proportion of circulating CD4^+^CD49d^hi^ T cells is an early biomarker of the severe form of GRMD. The detailed results are given in Table 1, and a graphical representation is provided in Fig. 2.

**Table 1.**
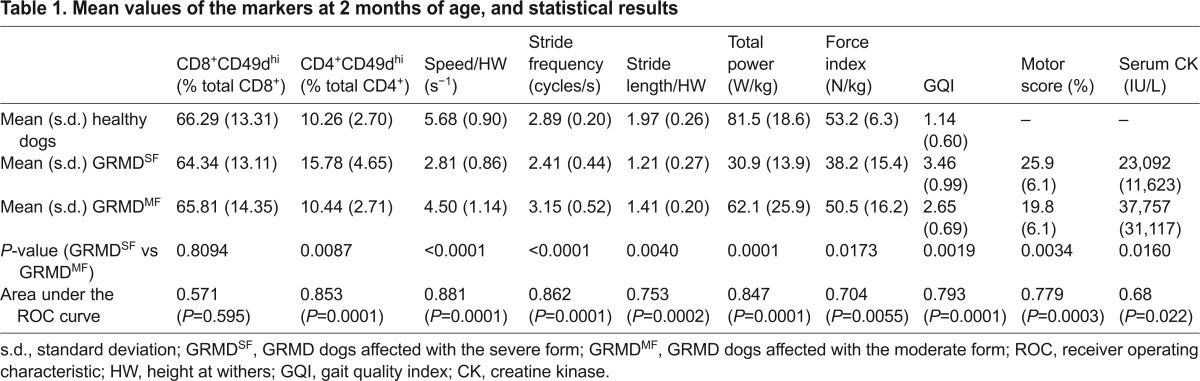
Mean values of the markers at 2 months of age, and statistical results

### Early gait abnormalities are more pronounced in GRMD dogs with a severe clinical form

During the gait test at 2 months of age, 23 dogs spontaneously selected to run at a trot, and 33 dogs to run at a bound gallop, while one dog walked. Seventy percent of dogs (*n*=30/43) that went on to develop the moderate form ran at a bound gallop, whereas only 20% of dogs (*n*=3/15) that went on to develop the severe form used this gait type. Therefore, the spontaneous use of the bound gallop at the age of 2 months can be considered as a good prognosis factor (χ^2^=12.48, *P*=0.0019).

Consistent with these gait differences, the dogs that would develop the severe form also ran significantly slower than dogs who would develop the moderate form (*P*<0.0001), mainly due to a significantly lower stride frequency (*P*<0.0001) associated with a shorter stride length (*P*=0.004). Accordingly, the total power was lower in dogs with the severe form (*P*=0.0001), as well as the force index (*P*=0.017). From a more global point of view, despite the absence of differences between dogs that would develop the moderate or severe form regarding regularity and three-axial power distribution, the gait quality index revealed that the gait was significantly more impaired in dogs with the severe form (*P*=0.002), in accordance with the motor score, which was significantly higher in these individuals (*P*=0.003). The serum creatine kinase (CK) values were slightly decreased (*P*=0.016) in the GRMD dogs that would develop the severe form of the disease, and which were already less mobile.

**Fig. 3.**
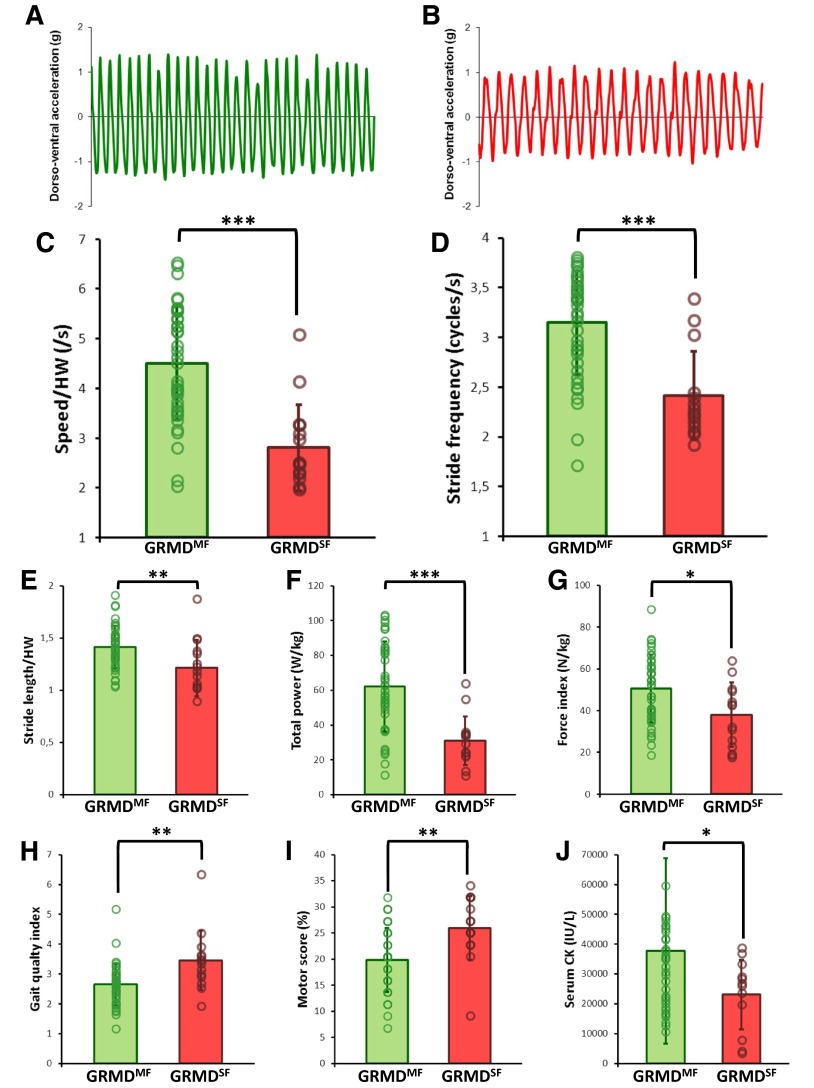
**Analysis of the motor function and results of the gait analysis, motor score and creatine kinase level in 2-month-old dogs as a function of their clinical form.** (A,B) A 10-second sample of dorso-ventral acceleration curves at a trot from 2-month-old GRMD dogs that went on to develop either the moderate (A; green) or severe (B; red) form of the disease. It should be noted that the frequency and amplitude of the curves are lower in the case of the GRMD dogs that went on to develop the severe form of the disease. (C–J) Graphs represent the comparison of the values obtained in 2-month-old GRMD dogs that went on to develop the moderate form (GRMD^MF^; in green) versus the severe form (GRMD^SF^; in red). The height of the histograms provides the mean and the error bars indicate ± 1 s.d. Individual values have been superimposed on the histograms and are represented by empty circles. Significant differences are represented by *P<0.05, **P<0.01 and ***P<0.001. (C) speed normalized by the height at withers (HW); (D) stride frequency; (E) stride length normalized by the height at withers (HW); (F) total power; (G) force index; (H) gait quality index; (I) motor score; (J) serum creatine kinase (CK).

Detailed results are given in Table 1, and graphical representations are provided in Fig. 3.

### Increased proportion of CD4^+^CD49d^hi^ T cells, decreased speed and lower stride frequency at 2 months of age are associated with an increased risk of early loss of ambulation

After adjustment for body mass index (BMI), number of blood leucocytes and presence of carpal transient contractures [a common clinical observation in young GRMD dogs that can impede locomotion (S.B. and I.B., unpublished data)], the proportion of CD4^+^CD49d^hi^ T cells at 2 months of age was linearly and significantly associated with time to loss of ambulation [adjusted hazard ratio (aHR) of 1.28 for a 1% increase; 95% confidence interval (CI), 1.05–1.57; *P*=0.02].

After the same adjustment, stride frequency at 2 months of age was linearly and significantly associated with time to loss of ambulation (aHR of 4.76 for a 1 second^−1^ decrease; 95% CI, 1.45–14.29; *P*<0.01).

**Fig. 4.**
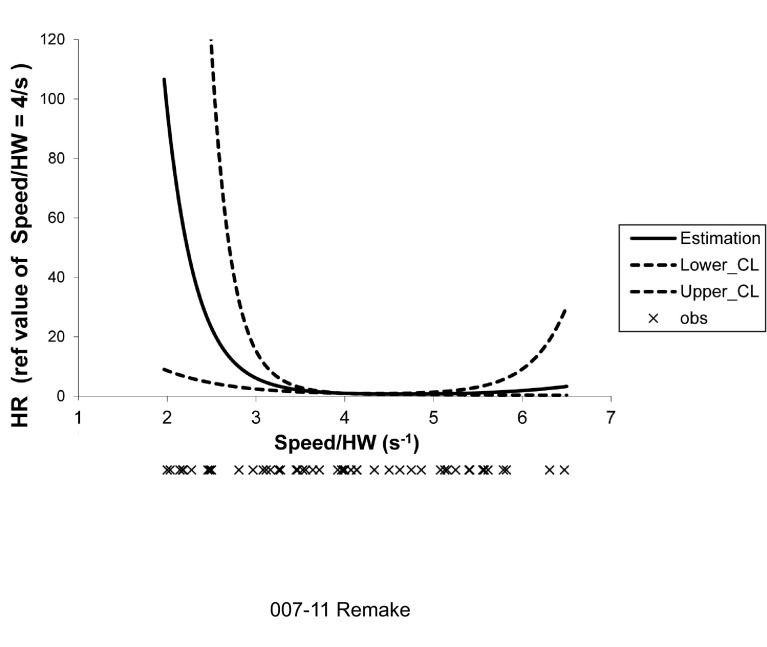
**Association between the age at which ambulation was lost and speed/HW at 2 months of age using a spline function with three knots.** Adjusted spline function analysis of the association between age at loss of ambulation and speed normalized by height at withers (HW), with three knots located at the 5th, 50th and 95th percentiles. This curve illustrates the very high hazard ratios at low speed/HW values. For example, dogs with a value of speed/HW of 2.5 second^−1^ have an increased risk for loss of ambulation of 17.70 (95% CI, 3.57–87.80; *P*<0.01) compared with dogs with a value of speed/HW of 4 seconds^−1^ (median of the whole population). The individual values of speed/HW are schematized under the graph (crosses). HW, height at withers; HR, hazard ratio; CI, confidence interval.

Again after the same adjustment, speed normalized by height at withers (speed/HW) was not linearly but significantly associated with time to loss of ambulation: dogs with a speed/HW value of 2.5 second^−1^ and dogs with a speed/HW value of 3 second^−1^ had an increased risk of loss of ambulation of 17.70 (95% CI, 3.57–87.80; *P*<0.01) and 5.28 (95% CI, 2.14–13.05; *P*<0.01), respectively, compared with dogs with a speed/HW value of 4 second^−1^ (Table 2; Fig. 4).

**Table 2.**
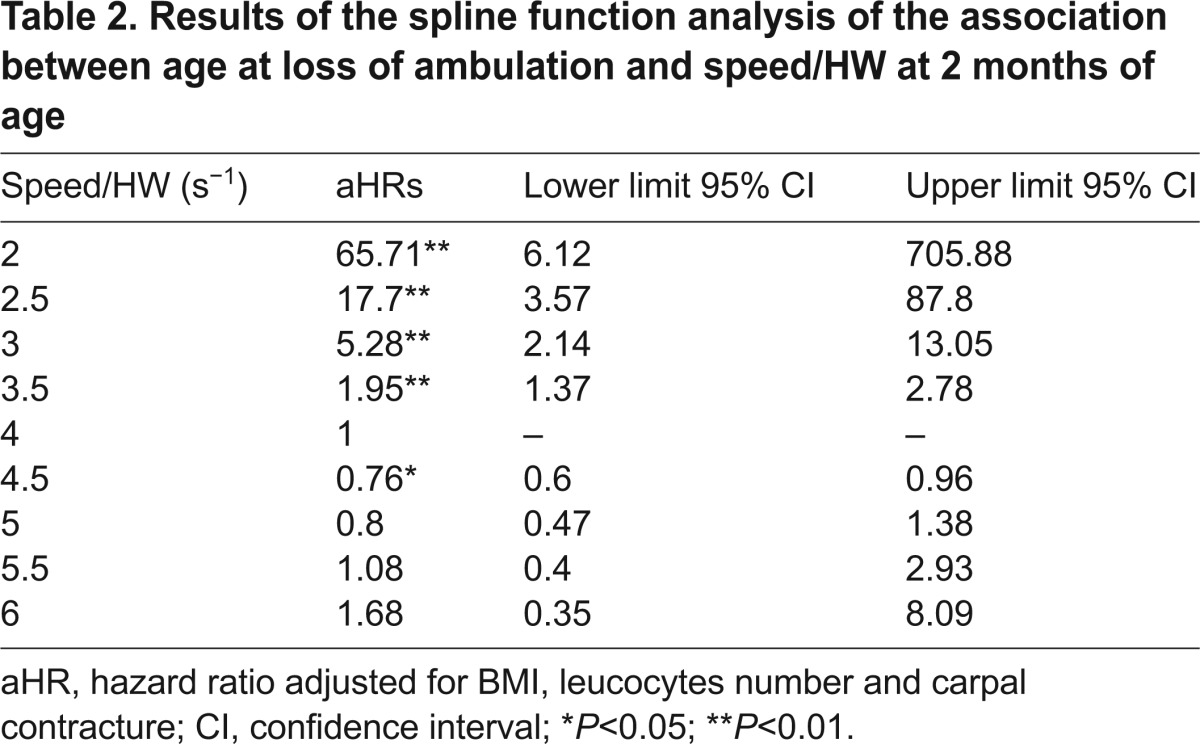
Results of the spline function analysis of the association between age at loss of ambulation and speed/HW at 2 months of age

### Predictive tests at 2 months of age can be proposed using the proportion of CD4^+^CD49d^hi^, the spontaneous walking speed and the stride frequency

In order to determine to what extent the lymphocyte and gait biomarkers could be used as predictive biomarkers for disease severity, and to assess the value of such a prognostic test, ROC (receiver operating characteristics) curve analyses were performed.

**Fig. 5.**
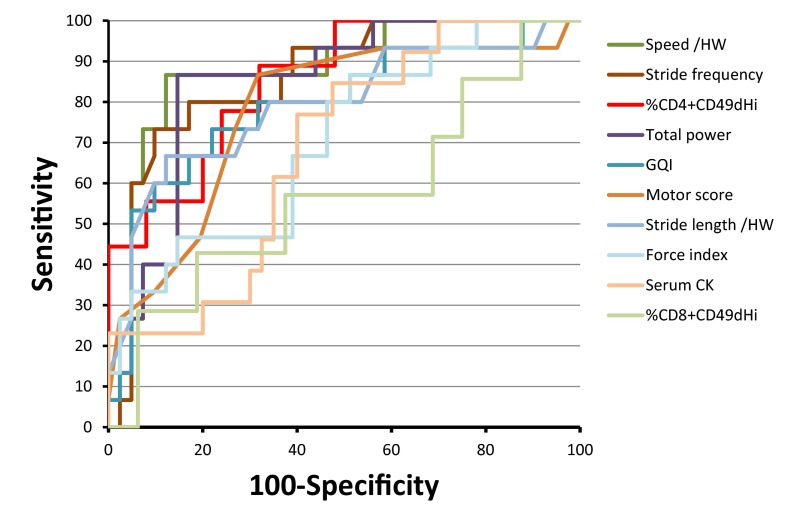
**ROC curves of the candidate markers for a predictive test.** The ROC curves of the ten parameters tested for their ability to predict severe forms are schematized on this graph. Each marker is represented by a specific color, indicated in the key on the right. Note that the curves with the highest areas under the curve (AUC) are those obtained using the speed (AUC=0.881) and the stride frequency (AUC=0.862). Another interesting point is that the percentage of CD4^+^CD49d^hi^ (AUC=0.853) remains highly specific (100%) until relatively high levels of sensitivity (44%). HW, height at withers; GQI, gait quality index; CK, creatine kinase.

Ten markers were tested: the proportion of circulating CD4^+^CD49d^hi^ and of CD8^+^CD49d^hi^ lymphocytes, the spontaneous speed and stride length normalized by the height, the stride frequency, the motor score, the gait quality index, the total power of gait, the force index and the serum CK activity. All the ROC analyses led to statistically significant results, except for the proportion of CD8^+^CD49d^hi^. The areas under the significant ROC curves ranged from 0.683 (serum CK) to 0.881 (speed/HW). The graphical representation of the ROC curves is shown in Fig. 5, and the results of the ROC curve analyses are given in Table 1.

According to the results of the ROC curve analyses, the use of these three combined markers can be proposed to predict the severe forms of GRMD as early as 8 weeks of age, each with interesting characteristics that can answer different needs of a study design. The speed/HW offers the best combination between sensitivity and specificity: by positioning a cutoff at 3.2759 seconds^−1^, the severe form (below this cutoff) can be predicted with a 86.7% sensitivity and a 87.8% specificity. The specificity of the test can be increased to lessen the risk of the prediction of false severe forms by choosing the use of the stride frequency: with a cutoff frequency of 2.44 cycles/s stride, the severe form can be predicted with a 90.2% specificity but only a 73.3% sensitivity. Finally the %CD4^+^CD49d^hi^ marker can be used with a 12% cutoff leading to a 88.9% sensitivity and 68.0% specificity. However this marker is of particular interest if a 100% specificity is required: since a 14.97% cutoff can be selected in this case, allowing a reliable selection of dogs with a predicted loss of ambulation, despite a large, but in some cases acceptable, proportion of undetected severe forms (44% sensitivity).

One of these three tests can be chosen, as a function of the requirements of the study design, so that it is possible to select either severe or moderate clinical phenotypes, to work on during preclinical trials. This should improve the analysis of the clinical outcome of treated dogs, by reducing inter-individual heterogeneity and increasing the significance of results.

## DISCUSSION

In this study, we have demonstrated that a set of carefully selected biomarkers can be used to predict the clinical evolution of GRMD dogs, the reference preclinical model of DMD.

Owing to the strong histological and clinical similarities shared with individuals with DMD, GRMD dogs have been shown to play a key role in the translational approach to develop candidate therapies for this disease. These canine counterparts of DMD individuals represent the ideal model to assess the efficacy of therapies at biochemical, histological and functional levels, in a pathological context very close to the human situation (Banks and Chamberlain, 2008; Bartlett et al., 1996).

The present study demonstrates that, by using these non-invasive biomarkers obtained at early stages of the disease, GRMD dogs can be separated into groups with slow or fast disease progression before their inclusion into a therapeutic preclinical trial, thus reinforcing the translational value of this animal model.

### Predictive biomarkers will improve the translational power of preclinical studies carried out on GRMD dogs

The novel predictive biomarkers we have identified offer the opportunity for a better handling of inter-individual heterogeneity, a prominent feature of GRMD dogs. Indeed, GRMD dogs are such a faithful model of DMD that they also reproduce the clinical heterogeneity seen in the human disease. However, in the context of an animal model, this can be seen as a major drawback when evaluating the effect of treatments, especially when the results are obtained in order to be directly translated to humans (Banks and Chamberlain, 2008). Recently, efforts have been undertaken to develop novel evaluation tools that allow a reliable functional evaluation of dogs, and are able to detect the effect of a treatment, despite this clinical heterogeneity (Barthélémy et al., 2012; Marsh et al., 2010; Thibaud et al., 2012). As a supplementary tool, the knowledge of the clinical status of the dogs at the beginning of the studies (i.e. possibility to divide dogs into groups with severe and moderate phenotype) would greatly increase the significance of the results and their translational value. One can even imagine selecting dogs upon their predicted evolution as an inclusion criterion. For example, only dogs with a predicted loss of ambulation before 6 months of age would be included, to evaluate the effect of a given treatment to prevent the loss of ambulation. In this case, the maintenance of the ability to walk would be a positive result per se.

Moreover, such a selection of the dogs by their predicted clinical profile would lead to homogeneous GRMD dog cohorts in preclinical trials, and thus reduce the number of dogs required to demonstrate a therapeutic effect, in the same way as it is envisioned in clinical trials by stratifying patients upon their genotype regarding modifier genes (Bello et al., 2012). This reduction in the number of dogs should lead to faster preclinical results and consequently a faster initiation of clinical trials. In conclusion, these predictive biomarkers could help to accelerate the translational process of therapies for DMD.

Moreover, it should be noted that the biomarkers we have presented in this study are ready to be used in predictive tests with known thresholds, sensitivities and specificities. They are reliable, easy and fast to obtain, and non-invasive (blood sample and gait test). Their use before the initiation of a potential treatment is thus a realistic proposal.

### These predictive biomarkers common to DMD individuals strengthen the translational value of the GRMD model

Another remarkable highlight of this study is the reinforcement of the already known similarities that exist between DMD individuals and the GRMD model. First, in a previous study we demonstrated that the proportion of circulating lymphocytes expressing high levels of CD49d is correlated with the severity of the phenotype in DMD individuals, and is able to reliably predict the age at which ambulation is lost (F.P.-M., W.S., S.D.S.-B., V.M., T.V., G.B.-B. et al., unpublished data). In this study, we report the same finding in GRMD dogs, at least for the CD4^+^ population. Secondly, the early gait abnormality that we have described is also very close to what has been described in DMD individuals. Indeed, it has been shown that the time to walk 30 feet, i.e. the spontaneous speed of gait, was strongly predictive of the time at which ambulation was lost (McDonald et al., 1995). Therefore, in both DMD individuals and GRMD dogs, the speed of walking can be used to predict the loss of ambulation.

The fact that both DMD individuals and GRMD dogs share the same predictive biomarkers is a new clue of the similarities existing between these two diseases and it emphasizes the interest of the GRMD model in translational research. Thus, GRMD dogs are not only similar to DMD individuals from many points of view, including clinical heterogeneity, but also their management during therapeutic trials can be very close to what is done for DMD individuals: similar evaluation tools have been developed for both species and this is now supported by reliable predictive biomarkers. Therapies targeting DMD can thus be preclinically tested in the same pathological and clinical context, using the same evaluation tools and the same biomarkers as controls of the ‘should-be’ clinical situation, optimizing the translation of results from the preclinical model to individuals with DMD.

### Limitations

Despite the step forward in preclinical trials for DMD provided by the identification of these new predictive biomarkers in GRMD dogs, this study also presents some limitations. First, because of the small number of dogs that were co-tested it was not possible to test the interaction that exists between lymphocytes and gait biomarkers. This should of course be done in future experiments, in order to propose a combined predictive test with an improved robustness, and also to better understand how these two features might interact. Indeed, even if gait modifications are complicated to decipher, and to link to a particular pathogenic pathway, because they result from a very global functional evaluation, they could originate from an enhanced muscle inflammation in severely affected animals, as suggested by the circulating lymphocyte biomarker. A more active inflammatory process in severely affected dogs, at least partly due to CD49d^hi^-expressing T-cell migration to muscles, could negatively impact ambulation either directly by inducing muscle pain, or by leading to increased muscle fibrosis, as described in affected individuals with more severe clinical presentations (Desguerre et al., 2009b). This aspect still remains to be investigated in GRMD dogs, using either muscle imaging and/or biopsies.

Furthermore, the objective of this work, which has been achieved, was to provide tools to improve the handling of GRMD dogs, but these biomarkers now have to be better understood from a mechanistic point of view. Further experiments will have to be carried out to explain the early modification of these biomarkers in dogs with severe forms of the disease. In individuals with DMD, it has been shown that a larger number of CD4^+^ and CD8^+^ CD49d^hi^ circulating cells are present in severely affected individuals; in comparison to the same subset of cells in healthy individuals, these cells in DMD individuals have an enhanced migration capacity, and are also a component of the lymphocytic population present in the muscles, where they probably contribute to the deleterious inflammatory process (F.P.-M., W.S., S.D.S.-B., V.M., T.V., G.B.-B. et al., unpublished data). Preliminary data obtained on GRMD muscle biopsies show that CD4^+^CD49d^+^ cells can be found in the inflammatory infiltrate like in DMD patients, but a correlation to the level of circulating TCD4^+^CD49d^hi^ cells remains to be assessed. A characterization of the inflammatory process in GRMD muscles will have to be performed in the future and compared to what is known in DMD, to explain some differences with the human context; notably, why the expression of CD49d on CD8^+^ cells is not modified in the canine species.

### These predictive biomarkers could help to validate modulatory pathways of both the canine and human diseases

In response to these limitations, further experiments should focus on the mechanisms that underlie the modifications of these biomarkers in severely affected dogs and humans. In this context, the GRMD dog is a favorable model to study inter-individual heterogeneity, because all the dogs share the same mutation in the dystrophin gene, the same environmental conditions and the same clinical management. It is thus much easier to work on modulatory pathways of the disease in dogs than in humans. Further experiments, including *in vitro* migration assays and *in vivo* pharmacological blocking of CD49d, will maybe confirm the CD49d-driven inflammation hypothesis. This canine cohort should also be genotyped for genetic modifiers of the human disease severity (Flanigan et al., 2013; Pegoraro et al., 2011), the *LTBP4* and *SPP1* genes, in order to determine to what extent the canine situation is comparable to the human one. This genetic investigation would also represent the opportunity to study the potential link between the CD49d^hi^ T cells, SPP1 and LTBP4 biomarkers in the same individuals. This could help to confirm the presumed role of the TGFβ pathway in the modulation of disease severity (Bartolomé et al., 2003; Flanigan et al., 2013), or to link these biomarkers to other pathogenic pathways, paving the way for new therapeutic targets translatable from GRMD to DMD.

### Conclusion

This study, carried out on the canine preclinical model of DMD, has taken advantage of its well-known inter-individual heterogeneity, one of the numerous common features with DMD, to identify predictive biomarkers of disease evolution. Lymphocytic and gait biomarkers, common to DMD patients, were successfully found to be able to predict severe forms of the canine disease. This study enhances the already high translational value for DMD of results obtained on GRMD dogs, by reinforcing the similarities between dogs and humans affected with muscular dystrophies, and by providing new tools to overcome the issue of inter-individual heterogeneity and to improve the quality of preclinical trials involving GRMD dogs. Finally, these biomarkers represent new avenues to explore in order to better understand clinical heterogeneity, and an opportunity to identify major modulatory pathways in dystrophin-deficient disease.

## MATERIALS AND METHODS

### GRMD dogs

All procedures were approved by the common ethical committee of the ANSES, ENVA and UPEC (ComEth ANSES/ENVA/UPEC), under the approval number 11/01/11-07.

The GRMD dogs included in this study were housed in the facilities of the neurobiology laboratory of the Veterinary School of Alfort. They were genotyped before the age of 2 months, as previously described (Bartlett et al., 1996). Healthy littermates matched for both gender and age were used as controls for the cytofluorometric experiments. The GRMD dogs were part of a natural history study, and a regular clinical follow-up was carried out throughout their whole life. Over a period of 4 years, a total of 61 GRMD dogs were included in the study; they were all tested at 2 months of age, and categorized at 6 months of age upon their ambulation status: ambulant at 6 months of age (moderate form) versus non-ambulant at 6 months of age (severe form). Data regarding the age at which ambulation was lost, if this event occurred, were also collected.

### Blood sample

Of the 61 GRMD dogs included in the study, 35 underwent an 8 ml venous blood sample at 2 months of age and were compared with eight healthy littermates. Among these 35 GRMD dogs, 26 were categorized as moderate forms, and nine as severe forms at 6 months of age. At the time of sampling, the dogs were checked for concurrent infections and, particularly, aspiration pneumonias. A blood cell count was also performed, as well as a biochemical assessment including a serum creatine kinase (CK) activity measurement.

### Cytometry

For cytometry, we used fluorochrome-labeled monoclonal antibodies with specificities for CD3, CD4, CD8 (Serotec, Kidlington, UK) and CD49d (Pharmingen/Becton Dickinson, San Diego, CA, USA). Isotype/ fluorochrome-matched unrelated antibodies were obtained from Pharmingen/Becton Dickinson. Peripheral blood mononuclear cells (PBMCs) from GRMD and healthy controls were isolated through ficoll-histopaque (Sigma-Aldrich, St Louis, MO, USA) sedimentation, using freshly obtained samples. PBMCs were first incubated in 96-well plates – with 5% fetal calf serum for 20 minutes at 4°C – and then subjected to fluorochrome-labeled primary monoclonal antibodies for 30 minutes. After washing, cells were fixed and acquisition for flow cytometry was carried out using a LSR II^®^ flow cytometer (Becton Dickinson, San Jose, CA, USA) equipped with FacsDiva software. A cell gate excluding cell debris and nonviable cells was determined using forward versus side scatter parameters. Analyses were done after recording 20,000 events for each sample, using the FACS Diva software.

### Functional motor assessment

Among the 61 GRMD dogs included in the study, 57 2 month-old GRMD dogs underwent a locomotor evaluation encompassing a clinical motor score, as well as a 3D-accelerometry test. Among them, 15 were then classified as severe forms owing to a loss of ambulation before 6 months of age.

The clinical motor score was performed using a previously described scoring grid (Barthélémy et al., 2012; Thibaud et al., 2007), containing 11 items; the higher the score, the more severe was the motor phenotype. The motor score was expressed as a percentage of the maximal score.

The 3D-accelerometry test was performed as previously described using a dedicated device (Locometrix^®^, Centaure Metrix, Evry, France) intending to record three-axial accelerations near the center of gravity during spontaneous gait (Barthélémy et al., 2009). Ten previously described variables were calculated from the acceleration curves in the software Equimetrix^®^ and are as follows: the speed (m/s), the stride frequency (cycles/s), the stride length (m), the regularity, the dorso-ventral, craniocaudal and medio-lateral powers (W/kg), the total power (W/kg), the force index [expressed in N/kg body weight, and calculated by normalizing the total power (W/kg) by the speed (m/s)], and a gait quality index recapitulating the association of seven variables. The speed and stride length, because directly influenced by the dog size, were normalized by the height at the withers (HW, m), and the axial powers were expressed as a proportion of the total power (%).

### Statistical analyses

Univariate comparisons between severe and moderate forms were assessed using chi-square or Student’s *t*-tests for categorical (e.g. the gait type) or continuously distributed variables, respectively.

For each biomarker that was associated with loss of ambulation at 6 months (i.e. severe versus moderate form) with a *P*-value <0.05, a receiver operating characteristics (ROC) curve was used to evaluate the effectiveness of the biomarker for distinguishing dogs with the severe form from those with moderate form.

Survival analyses were performed to assess the association between time to loss of ambulation and three following exposure variables: (1) proportion of CD4^+^CD49d^high^ T cells, (2) stride frequency, or (3) speed normalized by the height. Univariate and multivariate Cox proportional hazard models were used for each of the three exposure variables. The candidates for potential confounding variables were: body mass (kg), body mass index [BMI; calculated by dividing the body mass (kg) by the height at withers (m^2^)], presence of a carpal contracture, and blood leucocytes number. These last three candidates were associated with loss of ambulation with a *P*-value <0.20 in univariate analyses and were therefore included into each multivariate Cox model.

Spline functions (Desquilbet and Mariotti, 2010) were used to check the linearity assumption for each of the three exposure variables; this assumption was valid (i.e. *P*-value for a non-linear association >0.30) for the proportion of CD4^+^CD49d^high^ T cells and stride frequency, but not for speed normalized by the height (*P*-value for a non-linear association <0.01). In the latter model, a spline function with three knots located at the 5th, 50th and 95th percentiles was used for speed normalized by the height. For optimal adjustments, BMI as well as a leucocytes were both included using spline functions with three knots located at the 5th, 50th, and 95th percentiles (Desquilbet and Mariotti, 2010).

Analyses were conducted using Statistica (version 10, Stat Soft, Maisons-Alfort, France) and SAS^®^ V9.2 (SAS Institute, Cary NC) software. The level of significance was set at 0.05.
